# Assessing Without Words: Verbally Incomplete Utterances in Complaints

**DOI:** 10.3389/fpsyg.2021.689443

**Published:** 2021-09-27

**Authors:** Klara Skogmyr Marian

**Affiliations:** ^1^Centre for Research on Bilingualism, Department of Swedish Language and Multilingualism, Stockholm University, Stockholm, Sweden; ^2^Center for Applied Linguistics, Institute of Language Sciences, University of Neuchâtel, Neuchâtel, Switzerland

**Keywords:** verbally incomplete utterances, negative assessments, multimodality, vocalizations, complaints, conversation analysis, French interaction

## Abstract

This study investigates the use of verbally incomplete utterances in French-language complaints about third parties or situations. In these cases, a speaker initiates a turn with verbal means but stops talking before reaching lexico-syntactic completion. The utterance becomes recognizable as an expression of negative stance or as a precise negative assessment by virtue of the linguistic formatting of the turn-initiation, its position within the larger interactional context, and the speaker’s accompanying bodily-visual displays and vocalizations. Data consist of video-recorded coffee-break conversations among first and second language speakers of French. Using multimodal Conversation Analysis, the analysis documents recurrent linguistic formats of the verbally incomplete utterances and examines the interactional deployment of the utterances in two distinct sequential contexts: (1) in the initiation of complaints, and (2) at the end of complaint tellings or reports. In the first of these, the action of leaving a turn verbally incomplete and expressing stance with bodily-visual means allows the speaker to prepare the grounds for the complaint by foreshadowing the negative valence of the upcoming talk. In the latter case, the verbally incomplete utterance and accompanying vocal and/or embodied conduct are deployed as a summary assessment or upshot of the complaint which shows, rather than merely describes, the complaint-worthiness of the situation. In both cases, the utterances work to enhance the chances for the speaker to obtain affiliative responses from coparticipants. While prior studies on verbally incomplete utterances have suggested that such utterances may be specifically suitable for subtly dealing with delicate actions, in this study the utterances are sometimes produced as part of multimodal ‘extreme-case expressions’ that convey negative stance in a high-grade manner. The findings contribute to a better understanding of interactional uses of verbally incomplete utterances and of the multimodal nature of negative assessments. The study thereby furthers our understanding of how grammar and the body interface as resources for the accomplishment of context-specific actions and the organization of social interaction.

## Introduction

Assessment activities are highly multimodal in nature (Goodwin and Goodwin, 1987, 1992; Haddington, 2006; Lindström and Mondada, 2009). Speakers recurrently use prosody and bodily-visual conduct (gestures, facial expressions, changes in gaze direction and posture, etc.) to signal incipient assessments, modulate the strength of verbal stance expressions, and display their affective involvement in the activity (Goodwin and Goodwin, 1987, 1992; Ogden, 2006). Few studies have systematically investigated speakers’ use of bodily-visual conduct in assessing and responding to assessments, however (but see Ruusuvuori and Peräkylä, 2009; Kaukomaa et al., 2014; Park and Kline, 2020, and the discussion in Haddington, 2006). The present study contributes to bridging the research gap by examining a particular type of verbal-embodied stance displays in French interactions. In these cases, a speaker initiates a turn with verbal means but stops talking before reaching lexico-syntactic completion. Instead, the speaker offers bodily-visual conduct such as hand gestures or a combination of (non-linguistic) vocal and bodily-visual conduct that fills the slot of a projected assessment term, an entire clause, or signals turn completion (Li, 2016, 2019). The lexico-syntactic string is hearably incomplete on its own and instead made recognizable as part of a negative stance expression or a precise negative assessment with the help of vocal and/or bodily-visual conduct and based on the position of the utterance within the larger interactional context.

Excerpt 1 illustrates the phenomenon. In this excerpt, the university student Cassandra (CAS) complains about her current study situation, which involves having to write four ‘mini-essays.’ In line 2, Cassandra extends her turn (highlighted in gray) with *et c’est* (‘and it’s’). Instead of bringing the utterance to lexico-syntactic completion, she produces a small, barely audible vocalization (Keevallik and Ogden, 2020) and a depictive hand gesture (Streeck, 2009).

**EXCERPT 1:**
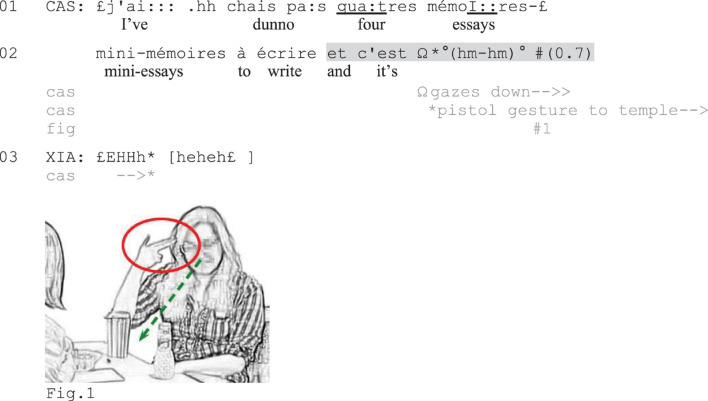
Mini essays.

Raising her hand toward her temple in a ‘pistol’ gesture (Figure 1), Cassandra figuratively (and non-seriously) expresses how she wants to shoot herself or be shot. The verbally incomplete utterance, completed by a conventional hand gesture, works as a negative assessment that conveys Cassandra’s strong negative stance toward her coursework. The recognizability of the utterance as a complete turn is seen in Xiang’s (XIA) laughing response in line 3 (for a more detailed analysis of this excerpt, see Excerpt 5 in Section “Summary Assessments of Complaint Tellings and Reports” below).

The study draws on the methodological tools of Conversation Analysis (CA) and Interactional Linguistics (IL) to conduct a systematic analysis of French speakers’ use of verbally incomplete negative assessments and stance expressions in a particular interactional activity: complaints about non-present third parties or states of affairs. The expression of negative stance is an integral part of complaints, deployed by complainants (speakers producing a complaint) to construct the ‘complaint-worthiness’ of the complained-about person or situation (Drew, 1998; Drew and Holt, 1988). Research on verbally incomplete utterances indicates that such utterances may be specifically suitable when speakers wish to convey negative stance toward a person or situation without verbally putting it ‘on record’ (Chevalier, 2008, 2009; Chevalier and Clift, 2008; Ford et al., 2012; Li, 2016, 2019; Park and Kline, 2020). In my data, this is not always the case. Instead, the verbally incomplete utterances perform stance expressions of varying degrees of affectivity and expressivity, from subtle ‘hints’ about the speakers’ stance to what I call ‘embodied extreme-case expressions’ of high-grade nature. By offering a systematic analysis of the use of verbally incomplete negative utterances in two different sequential contexts in complaints, the study extends prior research on both verbally incomplete utterances and on complaining in interaction.

The article is organized as follows: Section “Background” reviews existing literature on verbally incomplete utterances, (negative) assessments and stance expressions, and their role in complaint sequences. Section “Materials and Methods” presents the data and method used in the study. The analysis (Section “Analysis”) is divided into two parts. I first outline the formal composition of the verbally incomplete utterances and identify recurrent lexico-syntactic properties of the utterances in my data (Section “Formal Composition of the Multimodal Package”). I then analyze how these utterances are deployed in two specific interactional environments in complaint sequences (Section “Interactional Use in Complaint Sequences”), namely in complaint initiations and at the end of complaint stories or reports. I discuss the implications of the findings for our understanding of how grammar and the body interface as resources for the accomplishment of context-specific actions and the organization of social interaction.

## Background

### Verbally Incomplete Utterances and Bodily-Visual Completions

Human interaction is both intrinsically temporal and highly multimodal (e.g., Goodwin, 1979, 2000, 2007, 2013; Ochs et al., 1996; Mondada, 2014, 2018; Pekarek Doehler et al., 2015; Deppermann and Streeck, 2018; Keevallik, 2018). Because verbal and embodied resources have different temporalities (Deppermann and Streeck, 2018; Mondada, 2018), participants may deploy embodied conduct in socially meaningful ways both simultaneously with verbal resources and independently of these. A growing body of research has begun to document how speakers assemble verbal and embodied conduct into multimodal ‘packages’ for action (Hayashi, 2005; Goodwin, 2007; Iwasaki, 2009; Kärkkäinen and Thompson, 2018; Lilja and Piirainen-Marsh, 2019; Pekarek Doehler, 2019), ‘contextual configurations’ (Goodwin, 2000), ‘laminations’ (Goodwin, 2013), or ‘complex multimodal Gestalts’ (Mondada, 2014). Although these notions imply some variation in meaning, they all involve simultaneously and/or successively organized constellations of multiple semiotic resources that are put to use in locally contingent and socially meaningful ways. In this study, I focus on cases where bodily-visual conduct occupies the final slot of a turn-constructional unit (TCU) (cf. Olsher, 2004; Mori and Hayashi, 2006; Keevallik, 2013, 2014, 2018) and/or marks turn-completion (Li, 2016, 2019) after a verbally incomplete utterance, thereby making up a successively organized multimodal action package.

A verbally incomplete utterance may be described as an utterance that is initiated with verbal means but that is never brought to lexico-syntactic completion. Research on verbally incomplete utterances has shown that these are typically understandable to recipients despite their lexico-syntactic incompleteness, as seen in recipients’ relevant and normally well-timed responses (Hayashi, 2003, 2005; Olsher, 2004; Mori and Hayashi, 2006; Chevalier, 2008, 2009; Chevalier and Clift, 2008; Walker, 2012; Li, 2016, 2019; Lilja and Piirainen-Marsh, 2019). The recognizability of the turn ensues from various interactional clues, such as the design of the utterance-in-progress, its larger sequential context, and bodily-visual conduct offered both during and following the verbal components of the turn. The projection of actions and parts of actions (Auer, 2005, 2009) through various sequential, linguistic, and embodied means thus helps recipients anticipate what is coming next and facilitates mutual understanding and social coordination despite sometimes lexico-syntactically incomplete talk. Research on interactions involving second language (L2) speakers (Olsher, 2004; Mori and Hayashi, 2006; Lilja and Piirainen-Marsh, 2019) show that embodied completions of verbally incomplete utterances can be used effectively to ensure mutual understanding in interactions in which some interactants’ linguistic resources are (assumed to be) limited. Vocalizations and gestures are thus sometimes deployed to complete turns in lieu of verbal resources. Importantly, however, lexico-syntactically incomplete turns, whether completed or not by vocal or embodied means, recurrently occur in interactions between first language (L1) speakers too. My data confirm these observations, showing the use of lexico-syntactically incomplete turns in both L1 and L2 interactions.

Verbally incomplete utterances occur in a range of action contexts. Word-searches is a typical example, where speakers interrupt their ongoing turn as they have difficulties finding a particular word or expression and instead invite coparticipants to complete the search (e.g., Hayashi, 2003). Keevallik (2013, 2014, 2015, 2017) shows the recurrence of embodied TCUs or TCU completions in instructional demonstrations. Several studies also demonstrate the use of incomplete turns in the management of delicate issues and dispreferred actions (Chevalier, 2008, 2009; Chevalier and Clift, 2008; Ford et al., 2012; Li, 2016). Analyzing everyday French conversations, Chevalier (2008, 2009) and Chevalier and Clift (2008) show that speakers by leaving their utterance incomplete may accomplish socially delicate actions, such as informing about last minute changes or making a request, in a subtle way, putting these actions on record without verbalizing them. Li (2016) similarly demonstrates that speakers of Chinese use syntactically incomplete turns to accomplish negative assessments of third parties without uttering negative assessment terms. A recent study by Park and Kline (2020) on verbally incomplete negative assessments confirm these observations, while also documenting a particular recurrent lexico-syntactic format by which speakers accomplish critical assessments: utterances beginning with a neutral or positive clausal TCU followed by the contrastive conjunction *but* and a verbally incomplete clausal TCU.

Cumulatively, the research on verbally incomplete utterances shows that such utterances figure in different action contexts in both L1 and L2 talk and that they are unproblematic for intersubjectivity, whether completed by bodily-visual conduct or not (the latter confirmed by the telephone data examined by Chevalier, 2008, 2009 and Chevalier and Clift, 2008). In most cases, recipients respond to verbally incomplete turns with relevant next actions, thereby both displaying their understanding of the action performed by the turn and treating it as completed. Some of the abovementioned studies have documented speakers’ use of verbally incomplete utterances when expressing negative stance, and the next section elaborates on this by discussing research on negative assessments and complaining in interaction.

### Negative Stance Expressions, Vocalizations, and Complaining in Interaction

In social-interactional research, stance-taking typically refers to the publicly observable act of positioning oneself in a particular way vis-à-vis a stance object (Haddington, 2006). Affective stance-taking, in turn, refers to the public display of emotions (Goodwin et al., 2012). In the CA literature, stance-taking has since long been studied in the context of assessment activities.

Assessments involve speakers “evaluating in some fashion persons and events being described within their talk” (Goodwin and Goodwin, 1987: 6). Assessments can take the form of both activities and distinct actions. They occur with abundance in social interaction and are closely linked with epistemics: when a speaker offers an assessment, s/he “claims knowledge of that which he or she is assessing” (Pomerantz, 1984: 57; see also Heritage and Raymond, 2005). Assessment turns often take recurrent linguistic shapes. For English, Goodwin and Goodwin (1987, 1992) have noted the frequent assessment format [it] + [copula] (+ [adverbial intensifier]) + [assessment term], as in *it was (so) good.* For French, a corresponding, recurrent format is [ce] + [copula] + [assessment term], as in *c’est génial* (‘it’s great’), although often with a left- or right-dislocated assessable (Pekarek Doehler et al., 2015). As shown by Goodwin and Goodwin (1987, 1992), speakers tend to produce assessment segments (both the assessment term and any preceding intensifier) with marked prosody and embodied conduct that convey heightened involvement and that, together with the use of recurrent lexico-syntactic assessment formats, help coparticipants anticipate the upcoming assessment already early in the turn. There are few systematic investigations of the role of non-verbal resources in assessment activities, however. Some studies document how participants through gaze, pointing, and manipulation of objects may establish joint participation frameworks around assessments and the assessed objects (Haddington, 2006; Mondada, 2009). Facial expressions displaying stance can serve to stretch the temporal boundaries of assessments of stories and topic, offering different affordances for exchanges of affiliation than verbal resources (Ruusuvuori and Peräkylä, 2009; see also Kaukomaa et al., 2015, on how recipients’ facial expression may transform a speaker’s stance displays). Turn-initial frowns, in particular, have been observed to foreshadow different problems in interaction, including negative assessments (Kaukomaa et al., 2014). Prosody too serves as an important interactional resource in assessment activities, not only for projecting an upcoming assessment segment, but also in upgrading first assessments (Ogden, 2006).

Interactional research on vocalizations, or sound objects, has shown that these are deployed in systematic, socially situated ways to embody affectivity (Couper-Kuhlen, 2009; Reber, 2012) and perform evaluative work. Baldauf-Quilliatre (2016) has found that the frequently occurring vocalization *pf* in French always expresses affectivity in some way and in some cases also works as a negative assessment. Wiggins (2013) documents the use of vocalizations to embody negative food experiences, showing that disgust markers such as *eugh* are deployed both as self-standing assessments of the food and in combination with verbal assessment terms. Hoey’s (2014) work on sighing in interaction highlights the dual interactional potential of vocalizations as both markers of stance and as resource for the organization of turns and actions. The author shows that sighs produced before the onset of talk can serve to forecast negative valence and signal incipient dispreferred response, for example in the context of complaints. Sighs following a TCU may work as post-completion stance marker that signals the end of turns, while standalone sighs may serve to register and negatively evaluate a requested task. Finally, Hofstetter (2020) demonstrates the use of non-lexical moans to display negative affect in a playful, non-serious way. Together, these studies show that speakers deploy vocalizations as both full assessments and assessment segments, and sometimes use these for distinct interaction-organizational purposes. Crucial in this context is that the evaluative character of vocalizations only emerges in the local interactional context: it is the position within the sequence and the turn within the particular activity context as well as the prosodic and embodied delivery of the vocalization that make it recognizable as doing an assessment.

The present study is concerned with verbally incomplete utterances produced in the context of complaints about non-present third parties or states of affairs, also called *indirect complaints* (which can be contrasted with *direct complaints* that are about the recipient^1^). Although it is difficult to clearly define complaining (Edwards, 2005), prior research has highlighted the intricacy of this interactional activity and documented common characteristics of complaints. Indirect complaints are typically long sequences of actions in which speakers express strong negative stance about a ‘complainable’ to recruit affiliative or sympathetic responses from coparticipants (Drew and Holt, 1988; Drew, 1998; Traverso, 2009), often through affect-laden stories (Selting, 2012) or reports that exemplify (Günthner, 1995) and underline the severity of the situation, its ‘complaint-worthiness’ (Drew and Holt, 1988; Drew, 1998). At the same time, speakers have been observed to perform careful interactional work to attend to the delicacy of criticizing others, for example by introducing complaints in a stepwise manner that allows them to test the grounds for the complaint before launching the complaint fully (Traverso, 2009; Ruusuvuori et al., 2019). Some of the abovementioned studies on verbally incomplete utterances and vocalizations suggest that such resources may be specifically useful means to convey negative stance without verbalizing negatively valenced assessment terms (Chevalier, 2008, 2009; Chevalier and Clift, 2008; Ford et al., 2012; Wiggins, 2013; Baldauf-Quilliatre, 2016; Li, 2016, 2019; Park and Kline, 2020). The present study in part supports these findings, showing that verbally incomplete utterances may be deployed as resources for moving into complaining in a stepwise manner that delays explicit negative assessments (see also Skogmyr Marian, 2020, 2021). In addition, it identifies another use of multimodally completed assessments, namely as ‘embodied extreme-case expressions’ (cf. Pomerantz, 1986, on ‘extreme-case formulations’) that work as high-grade assessments (Antaki et al., 2000) that underline the severity of the complaint rather than as subtle criticism. As I discuss in Section “Summary and Discussion,” the discrepancy between my findings and prior research on verbally incomplete turns may be due to the participant framework (informal peer interactions) and the type of complaints analyzed in my data (which are often about inanimate matters), showing speakers’ use of multimodal stance expressions in recipient-designed and context-sensitive ways in complaint sequences.

In sum, research on stance-taking generally and assessments specifically has highlighted the multimodal nature of these activities and shown some of the ways in which prosody, gaze, facial expressions, and vocalizations may contribute to action-formation and interaction-organization in such environments. Further systematic analyses on the role of bodily-visual conduct and vocalizations in the context of verbally incomplete utterances used to express negative stance are nevertheless needed and may add some nuance to existing findings about the interactional purposes of such utterances.

## Materials and Methods

The main corpus used in the study consists of 89 sequences of indirect complaints produced by L2 speakers of French at elementary to advanced proficiency levels (from the corpus Pauscaf-L2, comprising 67 h of data). The collection of complaints was initially established for a longitudinal investigation of L2 complaint practices (Skogmyr Marian, 2020), based on key characteristics of indirect complaints identified in prior research. In brief terms, complaints were defined as interactional activities (rather than distinct actions) involving expressions of affective negative stance about non-present third parties, inanimate objects, or states of affairs that according to the speaker have affected him/her personally in an unfair or unreasonable manner. Many of the complaints concern inanimate objects or situations. Verbally incomplete negative assessments and stance expressions occur in complaints by speakers at all proficiency levels. The purpose here is not to investigate differences in use between speakers at different proficiency levels, but rather to expose recurrent characteristics of speakers’ use of verbally incomplete utterances across the data. As mentioned above, verbally incomplete utterances are recurrent features of both L1 and L2 interactions. To further support this claim, I have included a few examples from a corpus of interactions with L1 speakers of French (the corpus Pauscaf, comprising 10 h of data). Both datasets are based on coffee-break conversations between students taking place in university cafeterias at a university in the French-speaking part of Switzerland. These interactions were audio- and video-recorded (from two camera angles) by the author (Pauscaf-L2) and by collaborators and students at the university (Pauscaf). All participants have given their written consent to participate in the recordings and personal information has been anonymized in the transcripts. Images included in the analysis have also been anonymized based on the participants’ wishes.

The total collection comprises 47 lexico-syntactically incomplete utterances that express negative stance, of which the bulk (42) comes from the L2 corpus. Since no exhaustive analysis of verbally incomplete utterances expressing negative stance has been done in the L1 dataset and this corpus is considerably smaller than the L2 corpus (10 h vs. 67 h of L2 data), no quantitative comparison between the L1 and L2 interactions can be done and the L1 examples merely serve qualitative illustrative purposes. It seems safe to assume, however, that lexico-syntactically incomplete utterances are more common among speakers with limited linguistic resources in the language than among L1 speakers.

Many of the target utterances become recognizable as negative assessments of precise assessables. The broader category of ‘negative stance expression’ refers to examples in which the incomplete turn does not take a canonical assessment format, but in which the stance object is inferable from the interactional context. The data have been transcribed according to Mondada’s (2019) multimodal transcription conventions and analyzed sequentially based on the principles of CA and IL.

## Analysis

The first part of the analysis (Section ‘‘Formal Composition of the Multimodal Package’’) presents an overview of the formal composition of the verbally incomplete utterances in the data. This overview exposes recurrent features of negative assessment turns and stance expressions in spoken French that have not been documented in prior research, thereby contributing to the growing empirical evidence about the particularities of French grammar-in-interaction and its interface with multimodal resources. The second part of the analysis (Section ‘‘Interactional Use in Complaint Sequences’’) examines the interactional workings of the utterances in complaint sequences, by showing how speakers deploy verbally incomplete utterances to prepare the grounds for complaints or as summary assessments/upshots of complaint stories or reports^2^.

### Formal Composition of the Multimodal Package

The phenomenon under scrutiny may be described as follows: Through verbal means, the speaker sets up a projection (Auer, 2005, 2009) of a single or compound (Lerner, 1991, 1996) TCU, but stops speaking before reaching verbal completion. The verbal segment is followed by either embodied conduct or a vocalization and accompanying embodiment. Together, the assembly of verbal and non-verbal conduct becomes recognizable as an expression of negative stance, typically as a negative assessment of a specific assessable. Recipients normally show recognition of the action by responding relevantly to the utterance or by offering collaborative completions (Lerner, 1991, 1996). The speaker him/herself may also offer a recompletion of the turn through a verbal gloss, thereby ‘translating’ in verbal terms what was just expressed embodiedly (Keevallik, 2013).

**Excerpts (a–h)** exemplify the types of lexico-syntactic forms of the utterances found in the data, in their order of frequency from most to least common^3^.


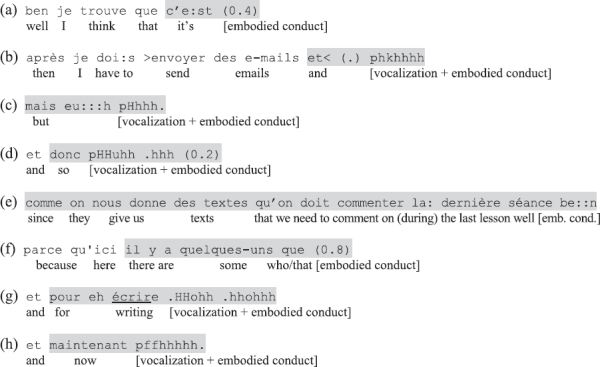


In (a), the neutral pronoun clitic *c’*, ‘it’ plus the copula *être* (‘to be’) in present or past tense is followed by a vocalization and/or embodied conduct. Here the neutral pronoun refers to a previously presented assessable, typically introduced with a left-dislocation or conveyed through a telling, which together with the verb projects an assessment term in the form of an adjective phrase. Quantifying adverbs like the French equivalents of ‘very’ (*très*) ‘really’ (*tellement, vraiment*), ‘a bit’ (*un peu*) recurrently precede the non-verbal conduct; negations ([*ne*] *pas*) as well as some particles similarly occur in some cases.

Examples (b–d) show the second most common type of utterance, namely one of the conjunctions *et* (‘and’), *mais* (‘but’), or *donc* (‘so’) – sometimes doubled as in *et donc* (‘and so,’ ex. d), plus vocal and/or embodied conduct. In these cases, the object to which the embodied stance expressions refer is not offered in the same TCU, but inferable from the larger interactional context. The conjunctions *et* (‘and’) and *donc* (‘so’) followed by embodied and/or vocal conduct sometimes occur as the last element of or following a listing, thereby working either to close the listing of negative elements or as a negative summary assessment of these (for an example with *donc*, see Excerpt 7 below). In contrast to the kind of ‘trail-off’ conjunctions documented by Walker (2012), the utterances here are typically *not* hearably complete immediately after the conjunction; instead, the multimodal packaging of the turn stretches past the syntactic structure and the simultaneous and successive bodily-visual conduct is recognizably part of the action, similar to the verbally incomplete turns ending with conjunctions documented by Keevallik (2017).

Much rarer types of verbally incomplete utterances include compound TCUs composed of a verbally initiated complex clause. In the cases initiated with a dependent clause (e), the first TCU makes a reference to the assessable and projects an independent clause about this referent to follow. In the examples initiated with an independent clause (f), a referent followed by a relative pronoun similarly presents a referent and projects more about this referent to follow. In both cases, instead of offering the second part of the compound TCU in verbal form, the speaker completes the projection with a vocalization and/or embodied conduct.

Finally, a few utterances that are left incomplete after self-standing prepositional phrases (g) or time adverbials (h) occur in the data, but are also rare. Similar to examples (b–f), the degree of morphosyntactic incompleteness of these turn-initiations is high. I return to the issue of varying morphosyntactic (in)completeness in the discussion (Section “Summary and Discussion”).

Table 1 provides a quantitative overview of the lexico-syntactic formats of the verbally incomplete negative assessments/stance expressions found in the L2 data (corpus Pauscaf-L2).

**TABLE 1:** Linguistic formatting of verbal initiations of the verbally incomplete assessments/stance expressions observed in the L2 data.

Verbal initiation	No. of occurrences	Percentage
*C’est/c’était* ‘it is’/‘it was’	19	45%
*Et*/*mais*/*donc* ‘and’/‘but’/‘so’	14	33%
Dependent clause	2	5%
Independent clause + relative pronoun	2	5%
Time adverbial	2	5%
Prepositional phrase	2	5%
Other/unclear	1	2%
TOTAL	42	100%

*Note that some adverbs and negations occasionally occur primarily in the first two formats (e.g., très, ‘very,’ pas, ‘not’).*

The high recurrence of *c’est* (‘it is’) plus vocal/embodied conduct concurs with observations in prior research about the frequency of this construction in assessment turns in French (Pekarek Doehler et al., 2015). Critical verbally incomplete assessments initiated with the conjunction *but* have been identified as a recurrent pattern in English (Park and Kline, 2020), and the frequency of turns left incomplete after *mais* (together with other conjunctions) in my data thus shows the recurrence of this pattern in French too. Overall, the inventory of lexico-syntactic structures closely resembles the findings by Keevallik (2015), who documents verbally incomplete utterances in dance demonstrations in English, Estonian, and Swedish (see particularly the summary on p. 328).

In terms of the non-verbal component of the utterance, this sometimes constitutes a clear assessment segment that lexically and syntactically completes the projected TCU. In other cases, the embodied conduct does not serve any role in the syntactic structure of the utterance, but rather signals turn completion (see Li, 2016, 2019). Sometimes, we see a combination of the two. Recurrent bodily-visual conduct used to convey negative stance includes eye rolls, lateral headshakes, stretched out tongue, and certain depictive gestures (Streeck, 2009), often deployed in concert with changes in gaze and posture, while pragmatic gestures (Kendon, 2004) may be used to mark turn-completion. Vocalizations expressing negative affective stance such as sighs (Hoey, 2014) and *pf*-sounds (Baldauf-Quilliatre, 2016) are also frequent. The varying lamination (Goodwin, 2013) of different bodily-visual resources affects the degree of expressivity of the conveyed stance. Concrete examples of the multimodal packaging of the turns are provided in the next section, in which I analyze different interactional uses of verbally incomplete utterances in complaint sequences and demonstrate how they become recognizable as negative assessments and stance expressions in these activities.

### Interactional Use in Complaint Sequences

The analysis focuses on the use of verbally incomplete utterances in two different sequential environments in complaints: (1) in the initiation of a complaint, where the utterance foreshadows the upcoming negative talk before any verbal description of the complainable has taken place, and (2) at the end of a complaint report or story, where the utterance is deployed as summary assessment or upshot that retrospectively evaluates the preceding talk. These utterances account for 88% of the L2 cases, of which 36% belong to the first category and 52% to the second (12% of the utterances occur at other sequential places or are unclear cases). The analysis thus extends existing findings about the use of verbally incomplete utterances that express negative stance by considering what they do in two specific interactional contexts. In addition, the analysis documents a continuum of affective stance and engagement expressed through the verbally incomplete utterances and their non-verbal continuation, going from subtle hinting to ‘embodied extreme case expressions,’ something which has not been documented in prior research.

#### Indexing Stance in Complaint Initiations

When produced at the beginning of a complaint sequence, the verbally incomplete utterance prospectively indexes the valence of the upcoming negative talk. While some of these utterances are part of story-prefaces that prepare the grounds for an incipient negative telling (cf. Sacks, 1974; Jefferson, 1978; Berger, 2017; see Excerpt 3), not all utterances precede stories (see Excerpt 2, 4). Considering the contingent nature of complaints, which require coparticipant collaboration to come about (Traverso, 2009; Ruusuvuori et al., 2019), embodied displays of stance, whether produced as part of a verbal-embodied package or self-standing (for the latter, see Skogmyr Marian, 2020, 2021), seem to be a way for future complainants to ‘test the waters’ of their negatively valenced course of action before launching the complaint fully. If coparticipants respond affiliatively to the first stance display, the speaker can safely proceed with more explicit verbal criticism or other negative statements. This is what we see in Excerpts 2–4.

**EXCERPT 2:**
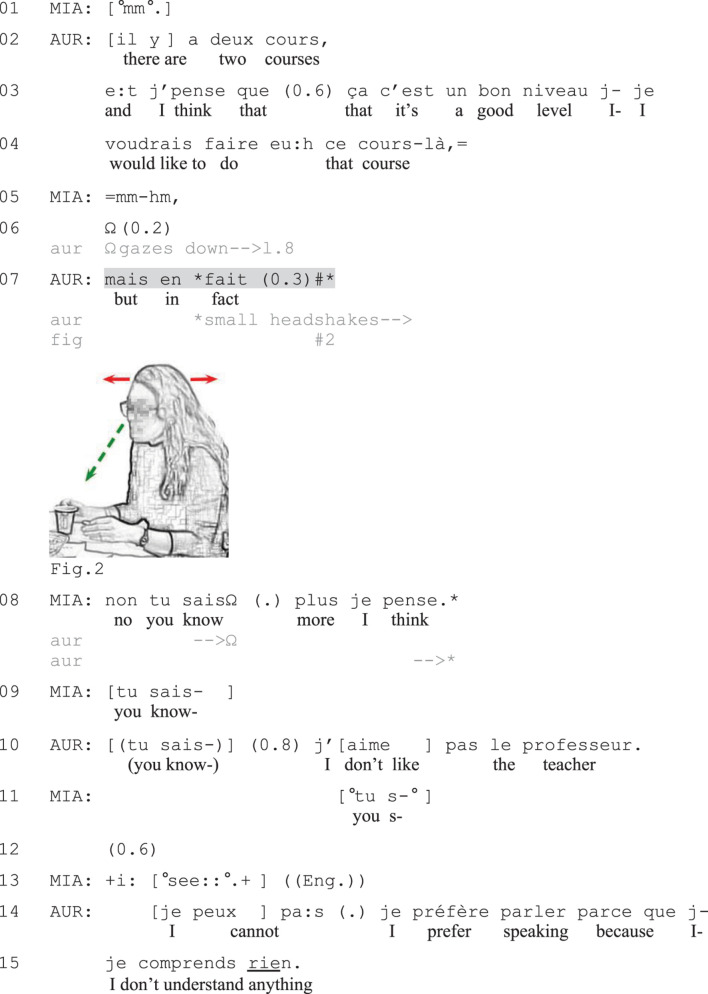
Level.

**EXCERPT 3:**
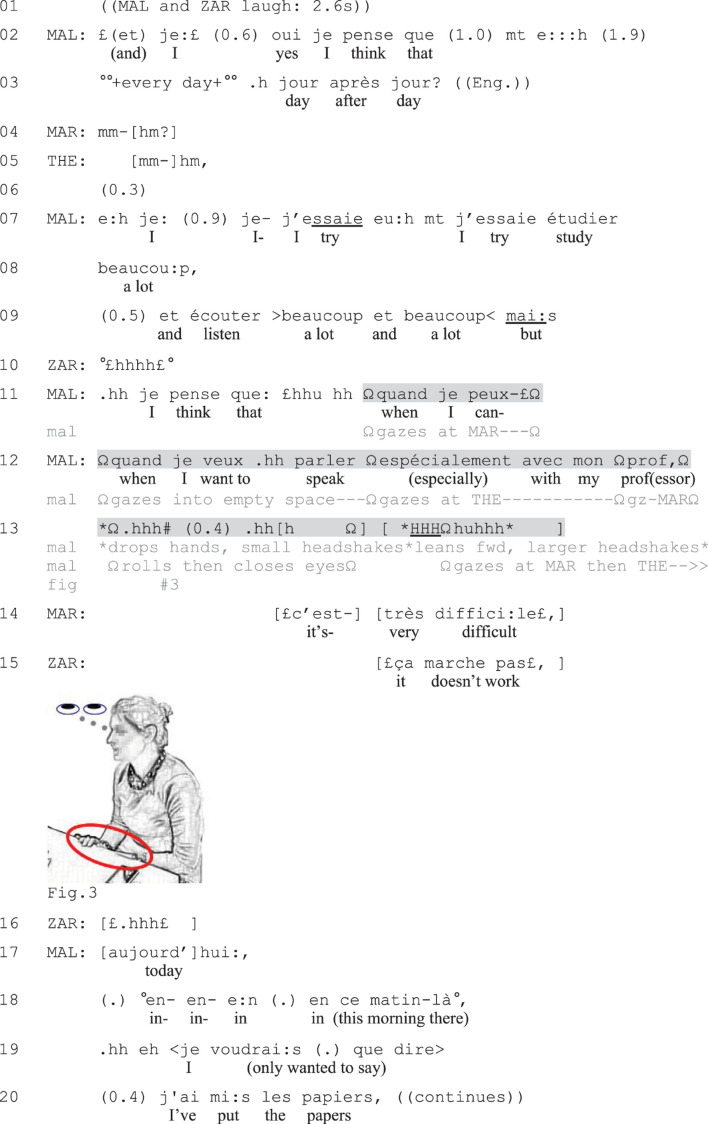
Speak.

**EXCERPT 4:**
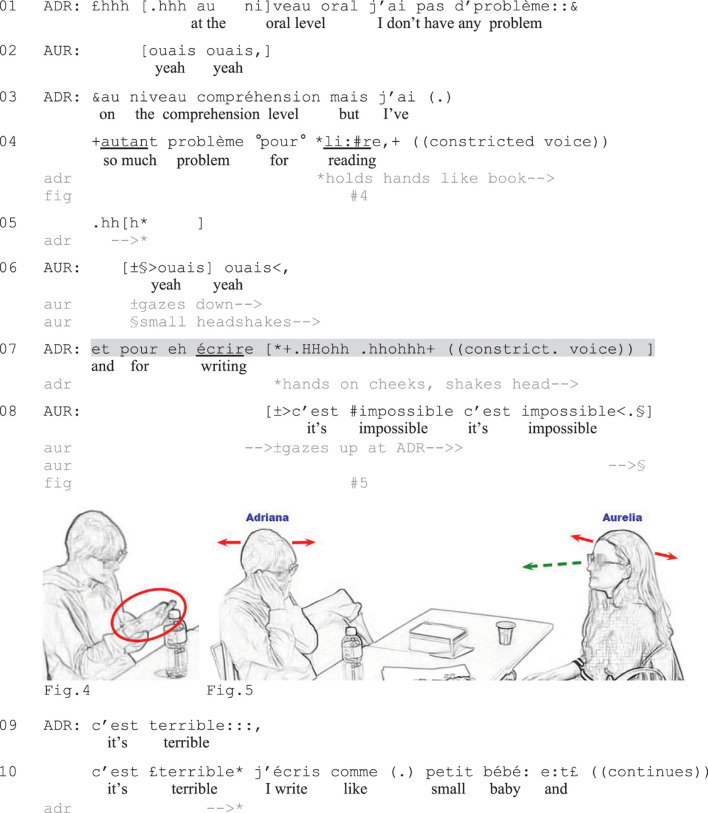
Writing.

The turns in this sequential position take varied lexico-syntactic formats and involve a continuum of affective loading. Whereas in some cases (Excerpt 2, 3), the non-verbal turn-continuations only hint at the negative valence of the upcoming talk, in other cases (Excerpt 4) they offer a high-grade, affect-laden expression. Common to all excerpts shown here is that the verbally incomplete turn is part of a contrastive formulation: a negatively valenced element that is introduced with the contrastive conjunction *mais* (‘but’) and stands in contrast with something of positive valence presented just before. Contrastive formulations have been observed as recurrent prefaces to both complaints and other potentially delicate actions (Sacks, 1992; Golato, 2005; Clayman, 2006). The following excerpts show that after a positive assessment or positively valenced observation, the speaker does not need verbalize the contrasting negative element to perform a recognizable negative stance expression (see Park and Kline, 2020, for similar observations on English data).

In Excerpt 2, Aurelia (AUR) will initiate a complaint about her previous French courses and a specific instructor. Before this, she reports on her course experiences; that she has already taken the introductory course and that there are two intermediate courses that she could now take. By assessing the intermediate courses as having a good level (line 3) and asserting that she would like to take those courses (line 4), Aurelia portrays an ideal situation of how she would like things to be. In the brief silence that follows Mia’s (MIA) receipt (lines 5), Aurelia lowers her gaze toward the table (line 6). She then initiates a contrastive formulation, which she does not bring to verbal completion (line 7).

The verbal string *mais en fait* (‘but in fact,’ line 7) projects a contrast to the ideal situation just described by Aurelia. At the production of *fait* Aurelia starts shaking her head slightly, her gaze still lowered (Figure 2), thereby producing a conventional embodied negation (Streeck, 2009). Instead of verbally completing the turn, she maintains the shaking during a brief moment of silence. Mia rapidly displays her understanding of Aurelia’s action as presenting a problem with the course by suggesting that Aurelia ‘knows more’ (line 8); that is, that the course is too easy for Aurelia. Instead of addressing this candidate account, Aurelia orients to another problem with the course, namely the teacher (line 10). She asserts that she does not like the instructor of the course (line 10) and she then starts accounting for this by offering negative observations about the course, namely that she does not understand anything (line 15) because the instructor focuses too much on written skills instead of speaking (not shown). Thus, in this excerpt the speaker provides a first, subtle hint at the upcoming complaint by offering an embodiedly completed negative stance expression (line 7). The syntactic format of the turn-initiation and its sequential position after a positively valenced assertion makes it recognizable as presenting a problem (Sacks, 1992; Park and Kline, 2020), and the coparticipant’s understanding of this is visible in her immediate response with a candidate reason for the problem (which however turns out to be wrong). The coparticipant’s turn works as an affiliative go-ahead signal for Aurelia to continue with negative talk (Traverso, 2009), which Aurelia does by expressing explicit criticism of the teacher.

Excerpt 3 includes a verbally incomplete compound TCU (Lerner, 1991, 1996). Here Malia (MAL) will initiate a complaint about her difficulties with speaking French (her L2) to her professor. She opens the sequence by situating her upcoming talk in time (lines 2–3) and her coparticipants confirm their listenership (lines 4–5). Malia then reports on her efforts studying French every day (lines 7–8). Doing so, she portrays herself in positive light before introducing a contrast with a prosodically emphasized *mai:s* (‘but,’ line 9). The contrastive formulation is composed of a bi-clausal turn initiated with *quand je veux parler espécialement avec mon prof* (‘when I want to speak especially with my professor,’ line 12). By gazing alternatively at her coparticipants during this part of the turn (lines 11–12), Malia works to further secure their attention to her talk.

The dependent clause initiated with *quand* (‘when’) strongly projects the delivery of an independent clause. Instead of offering the clause verbally, Malia drops her hands on the table, rolls her eyes (Figure 3) and breathes in, after which she utters a loud sigh (*HHHhuhhh*) and makes large headshakes, expressing her negative stance (Goodwin and Alim, 2010; Hoey, 2014) and a sense of exhaustion (line 13). The vocal and embodied conduct thus occupies the slot of the second part of the compound TCU. The coparticipants’ interpretation of Malia’s conduct as the expression of a difficulty is visible in their syntactically fitted collaborative completions (lines 14–15; see Lerner, 1991, 1996), which Malia accepts by initiating a telling about her difficulties (line 17 and onward). Like in the preceding excerpt, the coparticipants’ responses to the verbally incomplete utterance, here done through affiliative collaborative completions, ratify the speakers’ course of action and facilitate the development of the sequence into a complaint.

Excerpt 4 provides a final example of incomplete turns in complaint initiations. Before the excerpt, Aurelia (AUR) and Adriana (ADR) have exchanged compliments about each other’s spoken French, leading Aurelia to ask Adriana if she spoke French before coming to Switzerland. After confirming this, Adriana expands with a more extended answer, invoking the fact that she does not have any problem with speaking (line 1) or with oral comprehension (line 3). She then contrasts this with what she *does* have problems with, namely reading (line 4, Figure 4). After Aurelia’s agreeing response (line 6), Adriana expands with a verbally incomplete stance expression pertaining to her ability to write (line 7).

Adriana produces the prepositional phrase *et pour eh écrire* (‘and for writing,’ line 7) followed by two voiced in-breaths as she puts her hands to the sides of her face and shakes her head in a dramatic fashion (Figure 5). The lexico-syntactic formatting of the turn-initiation ties this segment back to Adriana’s prior turn (*pour lir:e*, ‘for reading’) and makes it recognizable as another problem area (for format-tying, see Goodwin and Goodwin, 1987). The prosodic realization, with strong prosodic stress on *écrire*, and the direct launch of the vocalizations make the non-lexical elements hearable as a continuation of the turn, part of the same prosodic unit as the verbal segment. Together, the verbal and non-verbal components of the turn construct a negative assessment of Adriana’s writing skills. In overlap with the vocalizations, Aurelia looks up at Adriana and offers *c’est impossible c’est impossible* (‘it’s impossible it’s impossible,’ line 8) in fast pace while still shaking her head (Figure 5). This negative assessment works as a collaborative completion (Lerner, 1991, 1996) by which Aurelia shows her alignment and affiliation with Adriana. Adriana then verbally glosses (Keevallik, 2013) her negative stance expressions through the high-grade negative assessment *c’est terrible* (‘it’s terrible,’ line 9), thereby upgrading the affective loading of the talk further. She subsequently develops the complaint by describing and illustrating how her writing resembles the writing of a small baby (line 10 and onward). In this case, the escalation of stance displays through the multimodally completed utterance and the verbal gloss is thus closely coordinated with the coparticipant’s expressions of negative stance, following Aurelia’s agreement token and small headshakes in line 6 and the affiliative negative assessment in line 8 (see Goodwin and Goodwin, 1987, 1992; Goodwin et al., 2012, on the coordination of stance and affect). Considering the topic of the interaction (the difficulty of learning French, an issue commonly discussed in the particular L2 setting) and the coparticipant’s supportive moves, the speaker can hence safely produce an ‘embodied extreme case expression’ of negative stance and ‘translate’ this in verbal terms before justifying it with an account.

Excerpts 2–4 have illustrated verbally incomplete utterances that become recognizable as negative stance expressions that foreshadow the valence and nature of the upcoming talk and thereby prepare the grounds for the impending complaint. The negative valence of the turn is recognizable based on turn-design and its multimodal delivery: In all excerpts analyzed here, the verbally incomplete utterance was part of a contrastive formulation initiated with a positive observation or praise and the contrastive conjunction *mais* (‘but’), similar to what has been observed by Park and Kline (2020) for English (see also Sacks, 1992; Golato, 2005; Clayman, 2006, for positive prefaces to negative statements more generally, and Keevallik, 2017, for embodied demonstrations initiated with contrastive conjunctions in English, Estonian, Finnish, and Swedish). All utterances were accompanied by multimodal displays of negative stance or affect. Recipients showed their understanding of the turns by responding relevantly to them. Doing so, they supported the negative talk and contributed to the development of the sequence, allowing the speaker to expand with more explicit negative criticism (Excerpt 2, 4) or initiate a complaint telling (Excerpt 3). In this sequential environment, verbally incomplete negative stance expressions thus provide a resource for speakers to show negative stance embodiedly/vocally and recruit coparticipants’ displays of alignment and affiliation with the speakers’ course of action before verbalizing criticism, thereby facilitating a move into complaining in a recognizable, stepwise manner (Traverso, 2009; Ruusuvuori et al., 2019; Skogmyr Marian, 2020, 2021).

The stance expressions vary between subtle embodied conduct (Excerpt 2), conventional vocalizations for the expression of negative stance such as sighs (Excerpt 3), and dramatic combinations of vocal and embodied conduct (Excerpt 4). While subtle stance expressions may be useful resources in specifically delicate situations (such as in complaints about specific individuals, cf. Excerpt 2), expressions with stronger affective loading are used when affiliative responses can safely be expected (as in Excerpt 4). To some extent, the observations thus support earlier claims about incomplete utterances deployed to avoid putting negative terms ‘on record’ (Chevalier, 2008, 2009; Chevalier and Clift, 2008; Ford et al., 2012; Li, 2016, 2019; Park and Kline, 2020), but this is hardly the case in the context of more high-grade multimodal displays of stance.

The lexico-syntactic formats of the turn-initiations also vary, but a common characteristic of the examples shown here is a high degree of lexico-syntactic incompleteness, whereby entire clauses or the copula are missing from the syntactic structure. As shown in the next section, this contrasts with the frequent occurrence of canonical assessment formats in summary assessments/upshots, where merely a projected adjective phrase is missing.

#### Summary Assessments of Complaint Tellings and Reports

Most verbally incomplete utterances in the data are done as summary assessments of the complaint (so far), produced after a verbal description of the complainable situation. Summary assessments, whereby speakers shift from description of events to assessment of these, are a common way for speakers to enter into the closing of stories and longer sequential units (Goodwin and Goodwin, 1987; Drew and Holt, 1988; Schegloff, 2007). Research on complaining has shown that complainants often deploy idiomatic expressions to express the gist of the complaint after a descriptive telling in pursuit of affiliative responses (Drew and Holt, 1988; Ruusuvuori et al., 2019). In my data, idiomatic expressions in complaint sequences are rare, and only used by the most proficient speakers. This is likely due to the documented difficulty involved in the learning of idiomatic expressions in an L2 (e.g., Forsberg, 2008; Erman et al., 2016), but perhaps also to the fact that idiomatic expressions seem to be less common closing devices in French than in for example English (Pekarek Doehler et al., 2011). The verbally incomplete summary assessments completed by vocal and/or embodied conduct seem to work in a similar way as idiomatic expressions, however, in that they convey the egregious nature of the just reported situation in a way that depicts rather than factually describes it. Almost all of these turns are left verbally incomplete after the SUBJ + COPULA structure *c’est/c’était* (‘it is/it was’; see Excerpt 5, 6) or the conjunctions *mais* (‘but’), *et* (‘and’) or *donc* (‘so,’ see Excerpt 7).

**EXCERPT 5:**
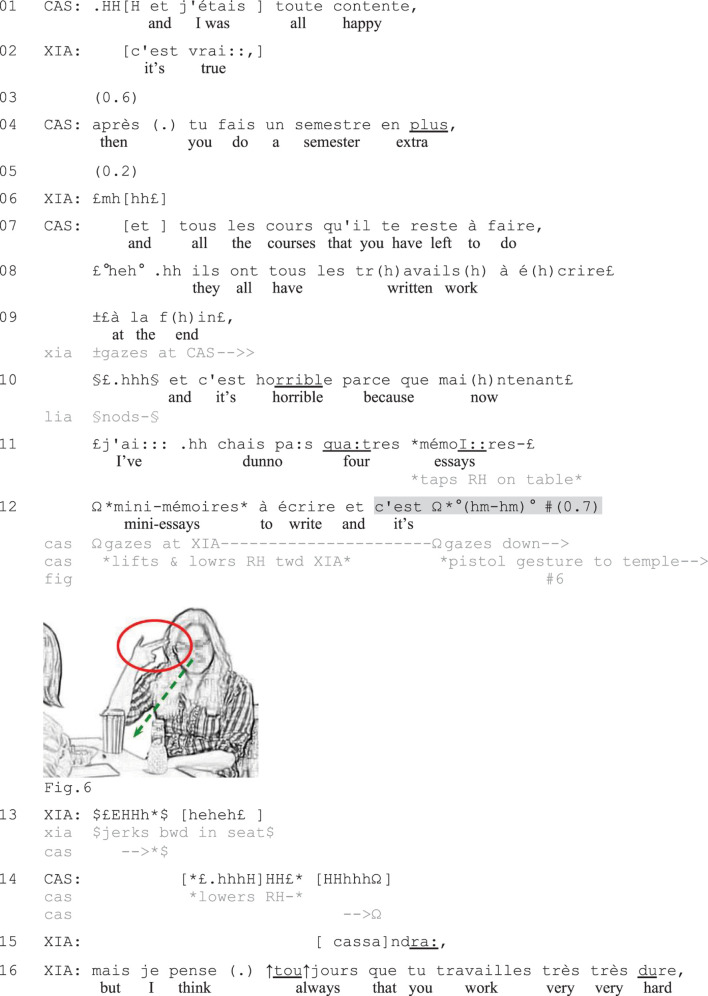
Mini essays.

**EXCERPT 6:**
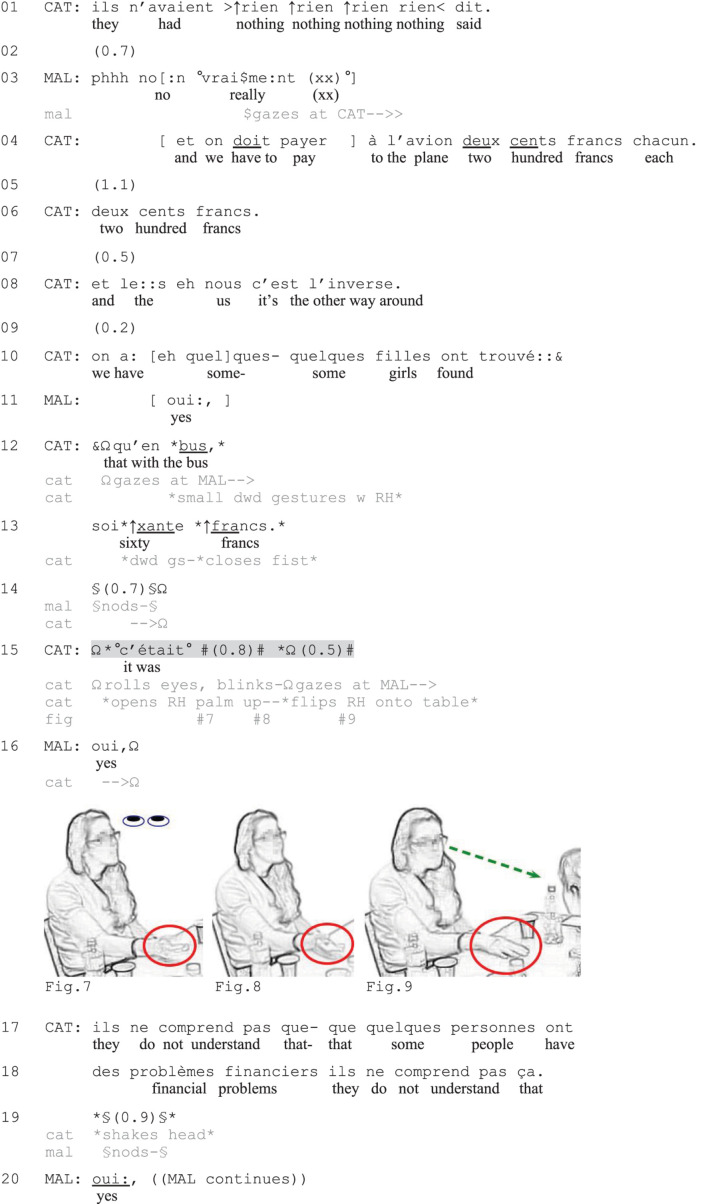
Sixty.

**EXCERPT 7:**
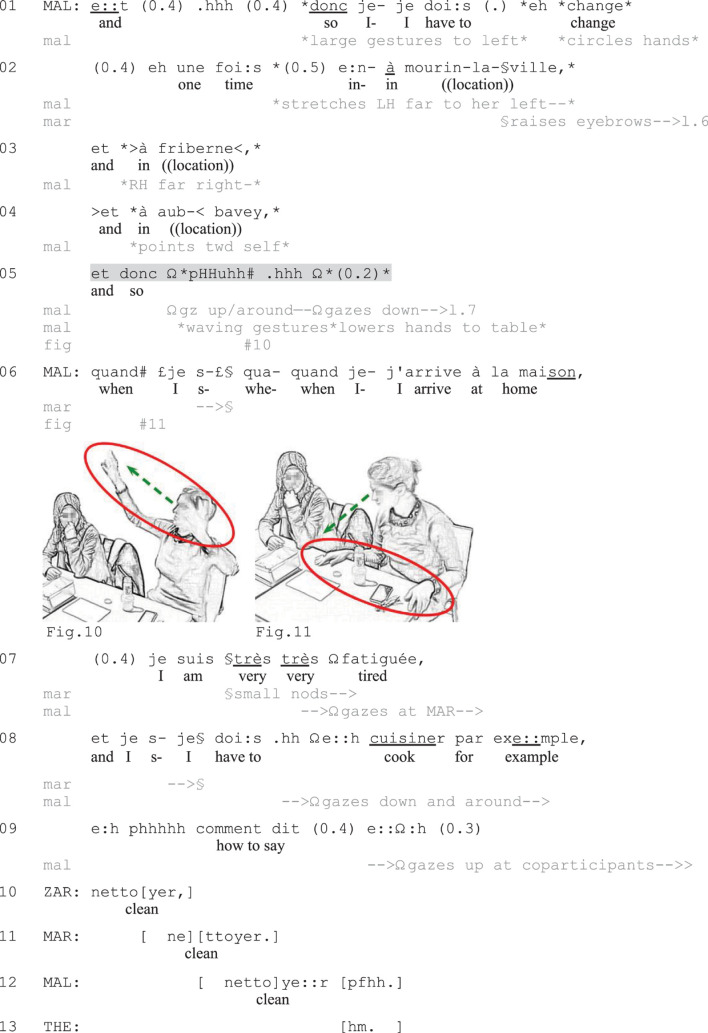
Change.

Excerpt 5, which presents a longer version of Excerpt 1 above, shows how participants may use verbally incomplete negative assessments to expressively mark the complaint story climax and invite coparticipants’ displays of affiliation. Before the start of the excerpt, Xiang (XIA) asked Cassandra (CAS) whether she is going away for the holidays, which Cassandra confirmed that she is not. To account for this, Cassandra started reporting how she during the first years of her bachelor program managed to avoid doing any writing assignments, which she was very happy about (line 1). She then contrasts this with her current situation (line 4), when she must take the remaining courses that all include written final assignments (lines 7–9), resulting in her now having four essays to write (lines 11–12).

Through extreme-case formulations (*tous les cours*, ‘all the courses,’ *ils ont tous* ‘they all have,’ lines 7–8; see Pomerantz, 1986) and a first high-grade negative assessment of the situation as *horrible* (‘horrible,’ line 10), Cassandra portrays her current situation as a strongly problematic one. To account for the horrible situation, she specifies that she now has four ‘mini-essays’ to write (lines 10–12). At the end of her account, she initiates what becomes recognizable as a summary assessment of the situation composed of *et c’est* (‘and it’s,’ line 12) followed by the depictive gesture of putting her right hand shaped as a pistol toward her temple (Figure 6) while gazing down. Doing so, she (jokingly) portrays the situation as something for which it is worth being shot (see Li, 2019, for the use of depictive hand gestures to complete assessments). Before producing the embodied assessment segment, at the mentioning of the key terms of the assessable (the essays that she needs to write), she taps her right hand on the table (line 11) and then lifts and lowers it toward Xiang while gazing at her (line 12) to ensure her attention to the upcoming talk. Xiang, who has been gazing at Cassandra since line 9, jerks back in her seat and bursts out in a laugh (line 13), thereby first responding to the humorous layer of Cassandra’s assessment. Cassandra, at the same time, lowers her hand and utters a voiceless sigh (line 14), pursuing the expression of negative affective stance (Hoey, 2014). Xiang then responds more seriously to Cassandra’s expressed troubles by trying to cheer her up through compliments on her study efforts (lines 15–16), while Cassandra insists on her difficult situation (not shown).

In this case, Cassandra’s verbally incomplete turn is recognizable as a negatively valenced summary assessment based on the sequential position of the turn, its lexico-syntactic format, and the use of a depictive gesture: Following several negative statements and the high-grade negative assessment *c’est horrible* (‘it’s horrible’) and an account, Cassandra’s second *c’est* projects another evaluative turn with similar valence. The use of a depictive gesture with clearly negative connotations also makes the assessment conventionally recognizable as a negative one. Similar to some of the verbally incomplete turns shown in Section “Indexing Stance in Complaint Initiations,” Cassandra’s utterance here can hardly be seen as a way to avoid putting negative terms ‘on record’. Instead, the expressive nature of the embodied conduct and the subsequent sigh make the turn recognizable as an animated story climax, which effectively recruits the coparticipants’ expressions of their appreciation of the story (signaled through laughter) and sympathy with the speaker (conveyed through positive assessments of Cassandra’s hard work). Through such multimodal climax, Cassandra laminates a humoristic layer onto the complaint story in a way that shows her troubles resistance (Jefferson, 1984; Edwards, 2005). The embodiedly completed summary assessment hence marks the ending of a complaint story in a depictive way that effectively recruits coparticipants’ displays of affiliation.

Excerpt 6 provides another illustration. In this case, the affect-laden work-up of the complaint makes a verbally incomplete turn recognizable as a negative summary assessment despite an only subtly expressed embodied turn completion. The complaint is a second complaint story (Selting, 2012) produced by Catarina (CAT) in response to a first complaint by Malia (MAL) about an expensive student trip. Catarina’s complaint is about the high costs associated with her own study program’s trip. In lines 1, 4, 6, she accuses the university staff of not having said anything about these costs and specifies that they had to pay two hundred francs each (approx. 200 USD) for the plane trip. Through extreme-case formulations, repetitions, and marked prosody, she expresses her strong negative affective stance toward the situation. In line 8, she expands the sequence by contrasting their situation to that of Malia’s, namely that in their case, the plane trip was more expensive than ground transportation (which was the other way around in Malia’s case). Catarina asserts that some girls had found bus tickets for merely sixty francs (lines 10, 12–13), that is, much cheaper than what they paid for the plane tickets. By means of prosodic emphasis and small downward hand gestures marking the stressed syllables, Catarina upgrades the strength and affective loading of her assertions (Selting, 2012), which work as the climax of the telling so far. As Malia merely nods in response (line 14), Catarina launches a summary assessment of the complaint, but she leaves her turn verbally incomplete (line 15).

Catarina initiates the summary assessment with *c’était* (‘it was,’ line 15) and simultaneously offers embodied conduct expressing negative stance. Having gazed at Malia before (lines 12–14), Catarina starts rolling her eyes and blinking rapidly while opening her right hand with her palm up (Figures 7–9), displaying her disapproval (Goodwin and Alim, 2010) and a sense of obviousness (Kendon, 2004). After 0.8 s of silence, she returns her gaze to Malia while flipping the hand onto the table, palm down (Figure 9), thereby embodiedly marking turn-completion and inviting Malia to respond (Stivers and Rossano, 2010). Malia, who has gazed at Catarina since line 3 and is leaning forward over the table closer to her (see Figure 9), immediately responds with *oui* (‘yes,’ line 16), claiming understanding of Catarina’s assessment. Catarina thereafter accuses ‘them’ of not understanding certain students’ financial problems (lines 17–18), thereby formulating in words the gist of her complaint. After a brief silence, in which Catarina continues to display her frustration with small headshakes and Malia nods in sympathy (line 19), Malia verbally expresses her affiliation (line 20 and onward).

In this excerpt, the verbally incomplete utterance is accompanied and followed by less prominent non-verbal conduct than in Excerpt 5 (no vocalization, no depictive gestures). However, the turn is still treated by the recipient as a complete assessment that is sufficient for reaching intersubjectivity. Catarina’s animated report and affect displays leading up to the turn, the status of the telling as a second complaint story (Selting, 2012), and the lexico-syntactic format (*c’était*, ‘it was,’ line 15) make the turn sufficiently recognizable as a display of negative stance. The low volume of the turn-beginning strongly contrasts with the preceding, prosodically upgraded TCU (line 13), and signals a move toward closing (Schegloff, 2007). The placement of the turn, following a silence in which the coparticipant merely nods (line 15), indicates that it is used as means to recruit more overt displays of affiliation (cf. Drew and Holt, 1988). The example resembles the type of incomplete turns observed by Li (2019) in which the non-verbal conduct following the incomplete verbal components (here the hand flip onto the table and gaze on recipient) does not substitute a specific lexical item, but instead signals the end of the turn. The eyeroll at the beginning of the turn, on the other hand, contributes to the multimodal packaging of the utterance as a negative assessment.

The final excerpt (Excerpt 7) exemplifies the use of a verbally incomplete negative utterance following a listing. This turn does not take a canonical assessment format but is left incomplete following the conjunction *donc* (‘so’). Here Malia is complaining about the long commute between her home and her workplace, and she lists the many train changes she needs to do on the way home (lines 1–4). With the help of large hand gestures to her sides, Malia animates the listing and underlines the extensive scope of her commute. After the third part of the list of locations (line 4), she initiates a summary statement (line 5), which she leaves verbally incomplete.

Malia completes the verbal initiation *et donc* (‘and so, line 5) with a sigh (*pHHuhh*) and large hand gestures high in front of her as she gazes up and around in different directions (Figure 10). The multimodal utterance embodiedly displays the overwhelming and burdensome nature of Malia’s long commute (conveyed through the gestures and wavering gaze) and the way it makes her very tired (expressed through the sigh). During a brief silence, Malia then lowers both her gaze and her hands, letting her hands fall on the table (line 5), marking the end of the TCU (Li, 2016, 2019). With her hands still on the table (Figure 11), she starts a new unit that works as a gloss (Keevallik, 2013) that elaborates the fatigue expressed through the vocally and embodiedly completed summary assessment (lines 6–7). Malia will then further expand her complaint in pursuit of more expressive recipient responses than what Mariana (MAR) has offered by raising her eyebrows in a display of astonishment (lines 2–6) and through small nods (line 7). She does so by exemplifying things she has to do when coming home (cooking: line 8), and Zarah and Mariana offer affiliative collaborative completions (Lerner, 1991, 1996) that contribute to the continued listing of chores (lines 10–12). In this excerpt, the verbally incomplete TCU thus marks the end of the listing activity, while also affectively expressing the consequence of the listed elements: The long commute with the many train changes is very tiresome for Malia. The sequential position of the turn following the listing, the conjunction *donc* (‘so’) which signals the formulation of a consequence, and the use of an affect-laden sigh (Hoey, 2014) all serve as resources for making the turn recognizable as a negatively valenced summary assessment or upshot of the complaint-so-far.

To sum up, in this section I have demonstrated how participants deploy verbally incomplete utterances as negative summary assessments that mark the end of complaint tellings or reports and that invite recipient displays of affiliation. In the first and the last case (Excerpt 5, 7), the assessments worked as highly expressive means to negatively summarize the situation described so far. The vocal-embodied turn-completions allowed the complainants not only to *describe* their stance to their coparticipants, but also to embodiedly *show* it. These uses are thus similar to Drew and Holt (1988; see also Ruusuvuori et al., 2019) observations about the recurrent use of idiomatic expressions to express the gist of a complaint, which offer evidence of the complaint-worthiness of the situation in a less refutable way than factual descriptions. The combination of embodied and vocal conduct results in a high-grade expression of affective stance, which works as an embodied extreme-case expression that helps construct the legitimacy of the complaint (cf. Pomerantz, 1986), underlining that it is worth complaining about. Excerpt 6 involved less expressive non-verbal conduct, which nevertheless displayed negative stance and marked turn-completion. In all cases, the sequential position of the utterances, following an often animated, affect-laden negative telling, was crucial for making these recognizable as summary assessments expressing negative stance. The use of routinized lexico-syntactic formats for the initiation of assessments (*c’est, c’était*) and conclusive statements (*donc*) similarly increased the projectability of the actions as offering summary assessments.

## Summary and Discussion

In this study, I set out to analyze the multimodal form and interactional use of utterances that are left verbally incomplete in French-language indirect complaints. I now summarize the findings, discuss their implications, and point out areas that deserve scientific attention in the future.

In terms of linguistic form, the lexico-syntactic formats of the incomplete turns to a high extent mirror the findings by Keevallik (2015) for English, Estonian, and Swedish, evidencing the projective force of these structures (SUBJ + COPULA clauses, contrastive conjunctions, first clause of complex sentences, etc.) across different languages. Most prominently, utterances left incomplete after *c’est/c’était* (‘it is’/‘it was’) and the conjunctions *et* (‘and’), *mais* (‘but’), and *donc* (‘so’) constitute the bulk of the occurrences (78% of all cases). The distribution of lexico-syntactic formats is not exactly equal across the sequential positions, however. In fact, as illustrated in the analysis and shown in Table 2, utterances occurring in complaint initiations vary proportionally more in their linguistic formatting than those used as summary assessments/upshots, which predominantly adopt the canonical assessment format *c’est/c’était* (‘it is’/‘it was’) (cf. Pekarek Doehler et al., 2015) or are left incomplete after conjunctions (together, these two patterns account for 91% of the cases in summary assessments/upshots, compared to 60% of the cases in complaint initiations).

**TABLE 2:** Linguistic formatting of verbal initiations of the verbally incomplete assessments/stance expressions observed in complaint initiations and as summary assessments/upshots (Pauscaf-L2; 5 occurrences of utterances used in other sequential environments have been excluded).

Verbal initiation	Initiations No. of occurrences (%)	Summary assessments/upshots No. of occurrences (%)
*C’est/c’était* ‘it is’/‘it was’	6 (40%)	11 (50%)
*Et*/*mais*/*donc* ‘and’/‘but’/‘so’	3 (20%)	9 (41%)
Dependent clause	2 (13%)	−
Independent clause + relative pronoun	1 (7%)	1 (4.5%)
Time adverbial	1 (7%)	1 (4.5%)
Prepositional phrase	2 (13%)	−
Other/unclear	−	−
TOTAL	15 (100%)	22 (100%)

A slight difference in the degree of morphosyntactic completeness can also be seen between the two sequential environments, whereby turns in complaint initiations more often than turns used as summary assessments/upshots leave whole clauses or the copula of a projected clause unspoken. The difference in linguistic formatting of the turns and thereby the degree of morphosyntactic completeness seems to correlate with the kind of interactional job the turns accomplish in their respective sequential positions: In the initiation of complaints, the speaker only hints at a negative situation that has not yet been detailed. Such hinting can be done in many different ways, and it may be that a principle of ‘less (talk) is more’ applies in these cases, leading the speaker to stop speaking early in the turn to leave the talk underspecified until coparticipants have given their go-ahead signals for more explicit negative talk. At the end of a complaint, the speaker’s affective stance has already been verbalized through various means and the summary statement is instead supposed to capture the gist of the reported situation. No ‘less is more’ principle is hence in place, and the use of a generic statement such as *c’était* (‘it was’) makes the turn-initiation highly recognizable as a summary statement referring back to the whole complaint. More research is needed to confirm these observations.

As for vocal and embodied conduct produced during and after the turn initiations, the analysis showed cases with embodied completions made up by the following features:

– depictive gestures (Streeck, 2009) that express precise semantic content (such as the ‘pistol gesture’ in Excerpt 1, 5);
– embodied conduct conventionally expressing negation or negative stance including lateral headshakes (Excerpt 2, 3, 4; see Streeck, 2009) and eye rolls (Excerpt 3, 6; see Goodwin and Alim, 2010);
– pragmatic gestures (Kendon, 2004) supporting interaction-organization such as the posing of hands on the table to mark turn-completion (Excerpt 6, 7);
– change in gaze and posture used both for action-formation and interaction-organization (e.g., gaze lowered vs. directed to coparticipant to embody exasperation or to invite recipient response);
– sighs (Excerpt 3, 7), guttural sounds on the in-breath (Excerpt 4), and other vocalizations (Excerpt 1, 5) that express negative affective stance (see Hoey, 2014, on sighing).

Although not exemplified in the analysis, the dataset includes turn-completions in the form of stretched *pf*-sounds, which are also associated with negative affective stance in French (Baldauf-Quilliatre, 2016), as well as guttural out-breath sounds (embodying vomiting) produced with a stretched-out tongue.

The strength and degree of affectivity of speakers’ stance expressions vary on a continuum from subtle hints to high-grade expressions of negative stance. Subtle hints may be accomplished through small headshakes (Excerpt 2) or eyerolls (Excerpt 6), hence conventional bodily-visual conduct for expressing negative stance used alone, without accompanying vocalization or other bodily-visual conduct. More high-grade expressions involve the lamination (Goodwin, 2013) of several semiotic resources such as sighing, gestures, eyerolls, headshakes, and facial expressions (Excerpt 4, 5, 7). Through such assemblies of bodily-visual and vocal conduct, speakers show a higher degree of affective involvement (Selting, 2012). The recognizability of the utterances as expressions of negative stance did not, however, seem to rely so much on the degree of expressivity of the embodied or vocal conduct or on their level of conventionality, since aspects of the turn’s placement in the sequence, its lexico-syntactic formatting, and the prosodic realization contributed to the recognizability of the actions. Instead, high-grade expressions of negative affective stance seemed to be used by speakers to underline the severity of the reported situation to solicit coparticipant engagement and displays of affiliation (similarly to what has been observed for extreme-case formulations, see Pomerantz, 1986). It may also be that bodily-visual and vocal completions allow speakers to express negative affective stance in a ‘richer’ way than through verbal assessment terms; a way that ‘shows’ rather than ‘tells’ (like Drew, 1998, and Drew and Holt, 1988, have argued is true for idiomatic expressions in English).

Besides conveying negative stance, the verbally incomplete turns served interaction-organizational purposes in the complaint sequences. At the beginning of a new sequence, they allowed the speaker to initially check the willingness of the coparticipants to support negatively valenced talk, thereby preparing the grounds for the subsequent development of the sequence into a complaint. At the end of a complaint or following a complaint story, the utterances marked the transition from the speaker’s longer turn to an exchange of affiliation with active participation from the coparticipants. While the same actions can surely be accomplished through verbally complete turns (cf. Drew and Holt, 1988; Drew, 1998; Traverso, 2009; Ruusuvuori et al., 2019), verbally incomplete utterances may be particularly apt for such interactional jobs as they can open up what Iwasaki (2009) calls ‘interactive turn spaces’ that invite co-participation before the turn has come to lexico-syntactic completion. Especially when coupled with response-mobilizing gaze (Stivers and Rossano, 2010; see particularly Excerpt 3, 6), the utterances hence provide structural affordances for affiliative responses (e.g., through collaborative completions) that contribute to the advancement of the sequence.

The findings about speakers’ use of what I call ‘embodied extreme-case expressions’ go against prior observations about the use of verbally incomplete utterances to manage delicate issues and dispreferred actions (Chevalier, 2008, 2009; Chevalier and Clift, 2008; Ford et al., 2012; Li, 2016, 2019; Park and Kline, 2020). Some verbally incomplete utterances with subtle stance expressions did occur in my data, and these might be specifically suited for introducing complaints in a careful way (e.g., Excerpt 2). But when speakers offer highly expressive multimodal turn-completions, they can hardly be seen as orientating to delicacy. The occurrence of embodied extreme-case expressions in my data seems to relate to several factors: (1) the placement of the utterance in the sequence, (2) the type of complaint, and (3) the participation framework. When a verbally incomplete assessment occurs at the end of a complaint, the complainant has already clearly conveyed negative stance and is no longer showing any orientation to delicacy (if such orientation was present at the beginning of the sequence). As seen in Section “Indexing Stance in Complaint Initiations,” high-grade stance expressions occur in sequence initiations too, however (e.g., Excerpt 4). This can likely be explained by the nature of the complainables and by the participant framework. Many complaints are about inanimate objects or situations, such as arduous course work or the difficulty of learning French, and the participants are university students chatting over a cup of coffee. Compared to complaints about specific third parties and complaints done in more formal environments (such as complaints about co-workers in workplace settings, see Ruusuvuori et al., 2019), most complaints investigated in this study are not oriented to with as much delicacy or dispreference. Complainants in my data can thus more safely express high-grade stance and expect affiliative responses, especially at the end of complaint sequences or when the coparticipants already early in the sequence reciprocates the speaker’s affective stance (as in Excerpt 4; see also Goodwin and Goodwin, 1987, 1992; Goodwin et al., 2012 on the collaborative coordination of stances). Future studies on other settings and participant frameworks would shed more light on the continuum of affective loading of embodiedly completed negative assessments.

A final note about the participants’ status as first or second language speakers. As convincingly shown in previous literature (Hayashi, 2003, 2005; Chevalier, 2008, 2009; Chevalier and Clift, 2008; Li, 2016, 2019) and demonstrated in some of the examples here (see Section “Formal Composition of the Multimodal Package”), verbally incomplete utterances are a regular feature of L1 interactions. It is likely that the relative distribution of verbally incomplete utterances differs between L1 and L2 speakers, however, and that non-verbal completions are particularly useful resources for speakers relying on a more limited repertoire of for example assessment adjectives and idiomatic expressions conveying negative stance (see Skogmyr Marian, 2020, on the development of negative assessment terms in the same L2 French dataset). As mentioned above, more research on L1 French is thus needed. In any case, the praxeological potential of the kind of multimodal packages investigated here stays the same, even if the frequency of use (and possibly the linguistic formats of turn-initiations) might differ between different populations.

In all, this study contributes to the growing interest within CA and IL to investigate the grammar-body interface in human interaction (see the contributions to this issue). More specifically, it contributes to the research on multimodality in turn-construction (e.g., Keevallik, 2013, 2014, 2018; Li, 2016, 2019; Lilja and Piirainen-Marsh, 2019) and in assessments (Haddington, 2006; Ruusuvuori and Peräkylä, 2009; Kaukomaa et al., 2014; Park and Kline, 2020) by providing a systematic analysis of verbally incomplete utterances in French-language complaining in interaction. Documenting both the multimodal form of the utterances and their interactional use, the study has shown that verbally incomplete negative assessments constitute a flexible interactional resource in complaint sequences. Followed by vocal and embodied conduct, the verbally incomplete utterances make out successively organized multimodal packages of action that speakers use to index different degrees of negative affective stance and accomplish context-sensitive social actions.

## Data Availability Statement

The datasets presented in this article are not readily available because the participants did not give written consent to share the data. Requests to access the datasets should be directed to KS.

## Ethics Statement

Ethical review and approval was not required for the study on human participants in accordance with the local legislation and institutional requirements. The patients/participants provided their written informed consent to participate in this study.

## Author Contributions

The author collected the main data corpus, carried out the empirical analyses, and authored the manuscript.

## Conflict of Interest

The author declares that the research was conducted in the absence of any commercial or financial relationships that could be construed as a potential conflict of interest.

## Publisher’s Note

All claims expressed in this article are solely those of the authors and do not necessarily represent those of their affiliated organizations, or those of the publisher, the editors and the reviewers. Any product that may be evaluated in this article, or claim that may be made by its manufacturer, is not guaranteed or endorsed by the publisher.

## Footnotes

1 Research on complaining has characterized direct and indirect complaints as quite distinct activities with considerable differences in their interactional management (see detailed discussion in Skogmyr Marian, 2020). To enhance comparability across cases, I have limited my focus to indirect complaints (for studies on direct complaints, see e.g., Dersley and Wootton, 2000; Monzoni, 2009; Kevoe-Feldman, 2018).

2 Not all complaints include prototypical storytellings with defined beginning and ends as outlined by Jefferson (1978) and Sacks (1974, 1992), among others. I use the term ‘report’ to refer more generally to participants’ detailing of a situation, which can be, but is not necessarily, part of a storytelling.

3 Examples (a), (c), and (e) show formats that occur in both the L1 and L2 data; these particular examples come from L1 speakers (corpus Pauscaf) whereas the remaining examples are from L2 speakers (Pauscaf-L2).
